# A Bayesian Approach to Real-Time Monitoring and Forecasting of Chinese Foodborne Diseases

**DOI:** 10.3390/ijerph15081740

**Published:** 2018-08-13

**Authors:** Xueli Wang, Moqin Zhou, Jinzhu Jia, Zhi Geng, Gexin Xiao

**Affiliations:** 1 School of Science, Beijing University of Posts and Telecommunications, Beijing 100876, China; wangxl@bupt.edu.cn (X.W.); interpreter_q@hotmail.com (M.Z.); 2 School of Public Health, Center of Statistical Science, Peking University, Beijing 100871, China; jzjia@math.pku.edu.cn; 3 School of Mathematical Sciences, Center of Statistical Science, Peking University, Beijing 100871, China; zhigeng@pku.edu.cn; 4 China National Center for Food Safety Risk Assessment, Beijing 100022, China

**Keywords:** Bayesian hierarchical model, foodborne disease, nowcasting, reporting delay, right truncation

## Abstract

Foodborne diseases have a big impact on public health and are often underreported. This is because a lot of patients delay treatment when they suffer from foodborne diseases. In Hunan Province (China), a total of 21,226 confirmed foodborne disease cases were reported from 1 March 2015 to 28 February 2016 by the Foodborne Surveillance Database (FSD) of the China National Centre for Food Safety Risk Assessment (CFSA). The purpose of this study was to make use of the daily number of visiting patients to forecast the daily true number of patients. Our main contribution is that we take the reporting delays into consideration and propose a Bayesian hierarchical model for this forecast problem. The data shows that there were 21,226 confirmed cases reported among 21,866 visiting patients, a proportion as high as 97%. Given this observation, the Bayesian hierarchical model was established to predict the daily true number of patients using the number of visiting patients. We propose several scoring rules to assess the performance of different nowcasting procedures. We conclude that Bayesian nowcasting with consideration of right truncation of the reporting delays has a good performance for short-term forecasting, and could effectively predict the epidemic trends of foodborne diseases. Meanwhile, this approach could provide a methodological basis for future foodborne disease monitoring and control strategies, which are crucial for public health.

## 1. Introduction

Patients with foodborne diseases often have a lack of awareness about the severity, which may cause them to delay seeing a doctor. Such kinds of delay may easily cover up the outbreak of food safety incidents, which is not conducive to the timely control of the disease outbreaks. Taking the occurred but not yet reported events into account, and tracking the true number of daily cases are essential to rapidly and accurately evaluate current epidemic trends. This stimulated us to study foodborne surveillance data to provide up-to-date information on the growth of the epidemic and current trends, so that from the local health department agency to the national public health agency or institute (e.g., the Center for Disease Control and Prevention in the USA, European Food Safety Agency, the European Centre for Disease Prevention and Control, etc.) can judge whether an outbreak is ongoing, assess the impact of control measures and implement capacity planning.

Previous studies on foodborne disease events have mainly focused on analyzing public surveillance data, estimating the actual incidence of foodborne disease in a country [1,2,3,4,5], and evaluating the burden of disease caused by various types of pathogens [6,7,8,9,10]. Heino et al. [11] proposed a randomized framework to identify the increasing number of foodborne disease events; Neill et al. [12] and Xiao [13,14] applied spatial statistical methods to explore the spatial aggregation of foodborne diseases. When diseases broke out in a certain area, the number of reported cases would surge correspondingly compared to the baseline data, which could be detected by anomaly pattern algorithms through numerical changes [15,16]. Guo et al. [17] designed a detection model of foodborne disease events and risk assessment by integrating the big-data of population, traffic, food production and sales and social media data under a spatio-temporal framework. The above studies ignore the reporting delay factor. However, the additional delays between onset date and reporting date in the public health surveillance database should not be ignored when processing tracking procedures. Lawless [18] firstly estimated the number of events that had occurred but not yet been reported and developed an approach to conduct robust predictions when incorporating random effects based on lately reporting data. Later, a robust algorithm to perform tracking procedures called “nowcasts” was used to correct for reporting delays [19]. Based on such concept, Hohle and Heiden [20] proposed a Bayesian nowcasting algorithm to deal with the short-term forecasting of the daily number of reported cases. Salmon et al. [21] improved the outbreak detection algorithms by taking reporting delays into account. Considering that the number of cases can increase dramatically in a matter of days in emerging food safety incidents, in this paper, we focus on how to make use of the daily number of visiting patients to forecast the daily true number of the patients, and we present how the proposed Bayesian nowcasting model could provide a more precise information of epidemic trends.

## 2. Data Material for Foodborne Diseases

**Figure 1:**
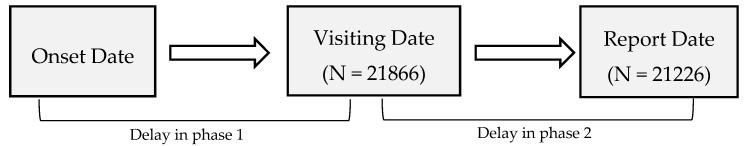
Illustration of reporting delay phases.

Our data, collected from the Foodborne Surveillance Database (FSD) of the China National Centre for Food Safety Risk Assessment (CFSA, Beijing, China), covered confirmed cases in all cities in Hunan Province, China, spanning a year from 1 March 2015 to 28 February 2016. Table 1 shows examples of our collected data information. Each confirmed case consists of symptom onset date (the self-reported date when the patient was attacked by a foodborne disease), and the visit date (when the patients went to see a doctor). The reporting delay is divided into two phases (Figure 1): Phase 1 is the delay between the onset date and visiting date; phase 2 is the delay between visiting date and the reporting date. In our data set, there are 21,866 patient visits and 21,226 reported confirmed cases.

Generally, the monitoring at the CFSA can be performed on time series aggregated by the date of symptom onset and the date of report arrival at the surveillance database. Specifically, till one day, we define “reported cases” as the currently available counts of patients confirmed by a doctor; and define “occurred cases” as the real number of patients attacked. The goal of nowcasting is to predict the true number of counts from the currently available counts.

**Figure 2:**
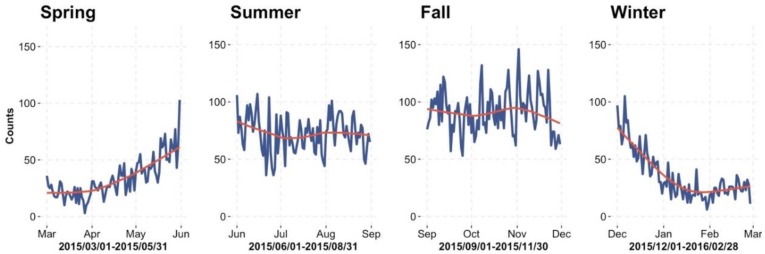
Seasonal trend of food-borne diseases during 1 March 2015 and 28 February 2016.

**Figure 3:**
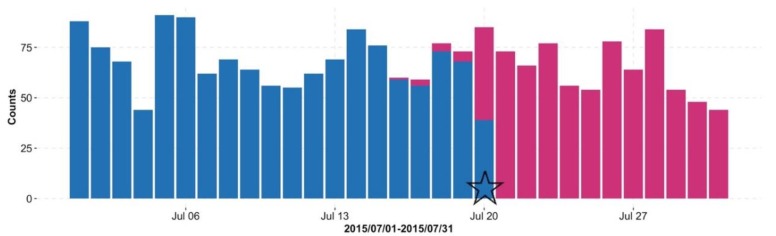
Daily number of “occurred cases” (red bar) with foodborne disease in retrospect. The blue bar denotes the number of available” reported cases” as of 20 July 2015 (indicated by the pentagram symbol).

Figure 2 plots the trend of foodborne disease, where the blue lines indicate the daily number of “occurred cases” and the red lines indicate the trend line. The curve of “occurred cases” starts to get into a high growth phase from May, and continues to peak till the beginning of November. From the end of November, the number of cases begins to decrease. Figure 3 shows the daily counts of “reported cases” (blue bar) and “occurred cases” (red bar); on 20 July 2015, 371 reported cases of 430 occurred cases (i.e., 86.3%, from 16 July to 20 July) have information available due to the reporting delay. As more reported data comes in, the number of reported cases (blue bar) is getting closer to the number of occurred cases (red bar) in Figure 3.

**Figure 4:**
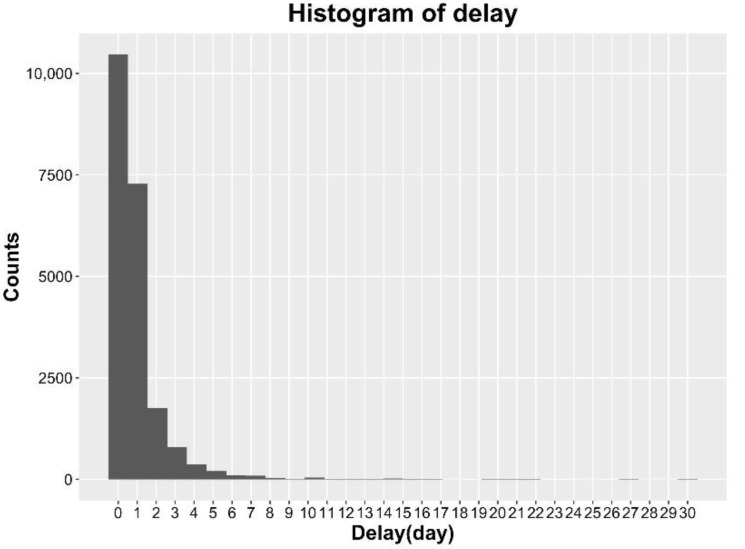
Histogram of the observed frequency of delays.

Figure 4 shows the histogram of observed frequency of delay on the basis of all cases, from which we see that many patients go to see a doctor on the first day when they have foodborne disease, more people have a few days’ delay and seldom have delays of more than one week. To facilitate understanding, we assume the maximum delay occurs up to 5 days due to the 3σ principle getting from the information in Figure 4. Note that the data will become less reliable and the information contained is not accurate when the delay becomes very large. We denote $p_{d},\;d=0,1,\ldots ,5$, as the observed proportion of the number of patients with delay d days to the total number of patients given a time span. Note that $p_{5}$ includes delay with days larger than or equal to 5 days, and $\sum_{d=0}^{5}p_{d}=1$.

**Figure 5:**
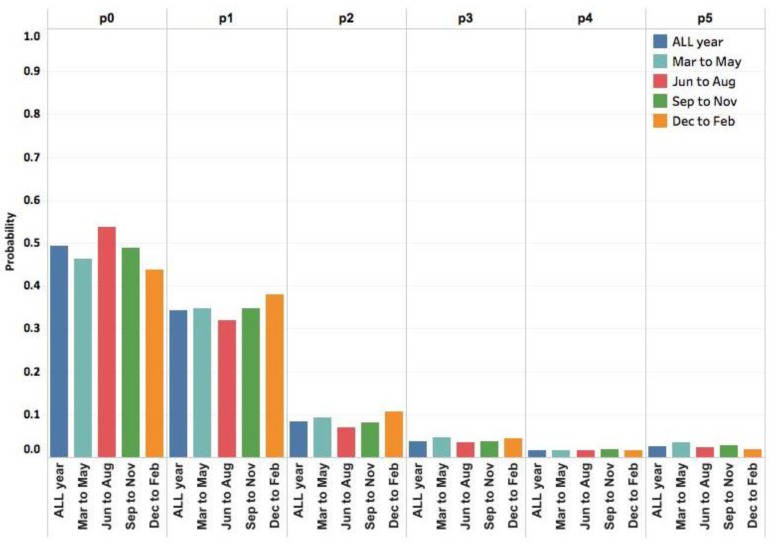
Seasonal distribution of delays. Here $p_{d},\;d=0,1,\cdots ,4,5,$ indicates the observed proportion of delay with d days. Category $p_{5}$ includes delay with days larger than or equal to 5 days.

From a seasonal perspective of delay distribution, Figure 5 suggests that the distribution of reporting delay does not change significantly with time, which therefore motivates us to assume that the patient behavior (corresponding to delay distribution) in a given region is stable with four seasons, if nothing else intervenes (e.g., policy).

## 3. Bayesian Nowcasting

Nowcasting is defined as the prediction of the present—the very near future or the very recent past. Recently, it has been regarded as a useful tool to real-time monitor the disease surveillance data [19,20], personal pro-health outdoor activities [22], and forecast the epidemics in public health settings [23]. In this paper, we develop a Bayesian nowcasting with truncation (BNT) approach to forecast the daily total number of cases in the presence of reporting delays, which can correct the current number of cases, thus to provide more accurate daily information on current epidemic trends. Below, we introduce necessary notation and assumptions.

### 3.1. Notation and Assumptions

We place our study in a discrete time setting where each unit represents a day. We use the notation of Lawless [18] to describe the prediction of the currently actual number of patients in the presence of delay. Let $n_{t,d}$ be the number of patients with foodborne disease at time t but reported with a delay of d days, which means that, these $n_{t,d}$ patients get foodborne disease at time t, but they arrive on hospital at time t+d. t takes values on {0, ⋯, T}, T denotes the current day or “now”, and d takes values on {0, ⋯, D}. Typically, one can assume that the maximum delay occurs up to D days since the data will become less reliable and the information contained is not accurate when the delay time d becomes very large. In our study, reports with a delay larger than D are included in the category of delay being “D days”. Note that when d>T−t, we could not know $n_{t,d}$, because at time T, the patients have not gone to hospital yet. So our data is right-truncated type of data. Formally, we devote $N_{t,T}≜N\left(t,T\right)=\sum_{d=0}^{\min\left(T-t,D\right)}n_{t,d}$ to be observed cases reported (those who go to hospital already) until time T. Thus with the limit of maximal delay, the real number of cases occurred at time t, 0≤t≤T, is:

$$N_{t}≜\sum_{d=0}^{D}n_{t,d}=\{\begin{array}{c} \sum_{d=0}^{D}n_{t,d},\;T-t\geq D,\;\mathrm{e.g.},\mathrm{full data}\\ \sum_{d=0}^{T-t}n_{t,d}+\sum_{d=T-t+1}^{D}n_{t,d},\;T-t<D,\;\mathrm{e.g.},\mathrm{truncated data} \end{array}$$

**Figure 6:**
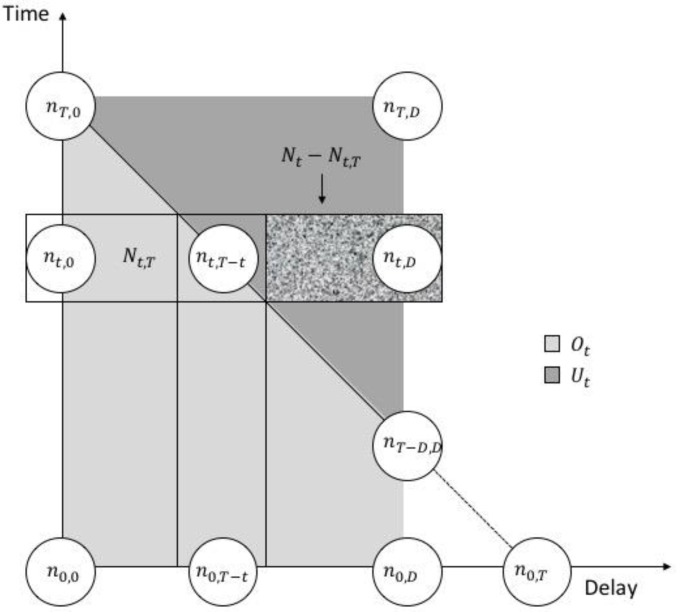
Data structure of our data and model. Available observations spanned by $n_{0,0},\;n_{T,0},\;n_{T-D,D}$ and $n_{0,D}$ are in the right-angled trapezoid $O_{T}$ while the triangle $U_{T}$ spanned by $n_{T,1},\;n_{T-D+1,D}$ and $n_{T,D}$ represents the cases that occurred but not yet reported. Delays greater than D are rare and ignored.

Note that $N_{t}$ is right-truncated for t larger than T−D. Our goal is to estimate the unobserved right-truncated $N_{t}$. We use Figure 6 to visualize the data structure. Each row indicates the total number of cases occurred at time *t*, the shaded box $N_{t}-N_{t,T}$ represents the cases that occurred but not yet reported. In order to facilitate model description, we divided the solution space into three parts: $A_{t}=\left\{\left(t,d\right):0\leq t\leq T,\;0\leq d\leq D\right\}$ covers all observed data and unobserved data while $O_{t}=\left\{\left(t,d\right):0\leq t\leq T,\;0\leq d\leq \min\left(T-t,D\right)\right\}$ contains observed $n_{O_{t}}$ only and $U_{t}=A_{t}\backslash O_{t}$ contains unobserved $n_{U_{t}}$ only. We draw them as the light gray trapezoid and as the darker gray triangle in Figure 6 respectively.

### 3.2. Predict the Distribution for $N_{t}$

Define $p_{d}$ as the probability that one patient will have a delay of d days. $p_{d}$’s should satisfy the following equation $\sum_{d=0}^{D}p_{d}$. With reference to Kalbfleisch and Lawless’s [24] and Zeger et al.’s [25] assumptions: the number of daily illness follows an underlying inhomogeneous Poisson process .t. $N_{t}∼Po\left(\lambda_{t}\right),\;t=0,\cdots ,T$. A reasonable data generating process is as follows:

$$N_{t}∼Po\left(\lambda_{t}\right)$$

$$\left(n_{t,0},\;n_{t,1},\cdots ,n_{t,D}\right)|N_{t}∼Multinomial\left(N_{t},\left(p_{0},\;p_{1},\;,\cdots ,p_{D}\right)\right)$$

where Po(⋅) denotes the Poisson distribution and Multinomial(⋅,⋅) denotes the multinomial distribution.

We try to infer the unknown $\lambda_{t}$ and $p_{d}$ parameters for t=0,⋯,T; d=0,⋯,D. Once these parameters are estimated, we could predict the distribution of $N_{t}$ given the observed information.

Using the Bayesian method, we assume that conjugate priors and the full hierarchical Bayes model could be described as follows:

$${\left(p_{D},\;p_{D-1},\;,\cdots ,p_{1}\right)}^{\prime }∼GD\left(\alpha ,\beta \right)$$

$$\lambda_{t}∼Ga\left(a_{\lambda },b_{\lambda }\right)$$

$$N_{t}|\lambda_{t}∼Po\left(\lambda_{t}\right)$$

$$\left(n_{t,0},\;n_{t,1},\cdots ,n_{t,D}\right)|N_{t}∼Multinomial\left(N_{t},\left(p_{0},\;p_{1},\;,\cdots ,p_{D}\right)\right)$$

where GD(α,β) is the generalized Dirichlet (GD) distribution with parameters $\alpha ={\left(\alpha_{0},\;\alpha_{1},\;,\cdots ,\alpha_{D}\right)}^{\prime },\;\beta ={\left(\beta_{0},\;\beta_{1},\;,\cdots ,\beta_{D}\right)}^{\prime }$. We denote $p≜{\left(p_{D},\;p_{D-1},\;,\cdots ,p_{1}\right)}^{\prime }$. $Ga\left(a_{\lambda },b_{\lambda }\right)$ as the Gamma distribution with parameters $a_{\lambda },b_{\lambda }$ which are known constants as the informative prior related to the morbidity. For this hierarchical model, the marginal (prior) distribution of $N_{t}$ is negative binomial distribution with the mean and variance as follows:

$$E\left(N_{t}\right)=\mu_{\lambda }=a_{\lambda }b_{\lambda }$$

$$Var\left(N_{t}\right)=\mu_{\lambda }+\frac{\mu_{\lambda }^{2}}{a_{\lambda }}$$

By application of Bayes theorem, we can predict the distribution of $N_{t},\;t=T-D,\cdots ,\;T$ given $N_{t,T}$ from the observed counts $N_{t,T}$:

$$\begin{array}{l} f\left(N_{t}N_{t,T}\right)\propto f\left(N_{t},N_{t,T}\right)\\ =\int \int f\left(p,\lambda_{t},N_{t},N_{t,T}\right)dp{d\lambda }_{t}\\ =\int \int f\left(p\right)f\left(\lambda_{t}\right)f\left(N_{t}|\lambda_{t}\right)f\left(N_{t,T}|N_{t},p\right)dpd\lambda_{t}\\ =\int f\left(\lambda_{t}\right)f\left(N_{t}|\lambda_{t}\right)d\lambda_{t}\int f\left(p\right)f\left(N_{t,T}|N_{t},p\right)dp \end{array}$$

From the above equality, we could calculate the posterior distribution of $N_{t}|N_{t,T}$ as follows:

(i) The integration $\int f\left(\lambda_{t}\right)f\left(N_{t}|\lambda_{t}\right)d\lambda_{t}$ could be computed by directly computing the density of negative binomial distribution $N_{t}$.
(ii) A Monte Carlo method could be used to approximate the second factor of the above equation. By this Monte Carlo method, we first sample p from generalized Dirichlet distribution, and then we compute the likelihood function and calculate the average of all data:
  $$f\left(N_{t,T}|N_{t},p\right)\propto {\left(\sum_{d=0}^{T-t}p_{d}\right)}^{N_{t,T}}{\left(1-\sum_{d=0}^{T-t}p_{d}\right)}^{N_{t}-N_{t,T}}$$

For example, when we calculate $f\left(N_{t}=nN_{t,T}\right)$, the Monte Carlo approximation is:

$$\int f\left(p\right)f\left(N_{t,T}|N_{t}=n,p\right)dp\approx \frac{1}{S}\sum_{s=1}^{S}f\left(N_{t,T}|N_{t}=n,p^{\left(s\right)}\right)$$

where $p^{\left(s\right)}$ is the sth sample from GD(α,β). Therefore, we can use the average to approximate $f\left(N_{t}=nN_{t,T}\right)$, for t=T−D,⋯,T and $n=0,1,\cdots ,N_{max}$, where $N_{max}$ is sufficiently large.

## 4. Main Results

### 4.1. Setup for Hyper-Parameters

We applied the Bayesian hierarchical model proposed above to the foodborne surveillance data time series of Hunan Province in China. To decide the hyper-parameter α,β in the prior of p and $a_{\lambda },b_{\lambda }$ in the prior of $\lambda_{t}$, we use the empirical Bayes ideas. Analyzing the observed data from 1 March 2015 to 28 February 2016, we found that the mean and variance of $N_{t}$ from the dataset are 57 and 982 respectively. Thus solving the following equations, we get the values for hyperparameters $a_{\lambda },b_{\lambda }$:

$$57\approx E\left(N_{t}\right)=\mu_{\lambda }=a_{\lambda }b_{\lambda },$$

$$982\approx Var\left(N_{t}\right)=\mu_{\lambda }+\frac{\mu_{\lambda }^{2}}{a_{\lambda }}$$

**Figure 7:**
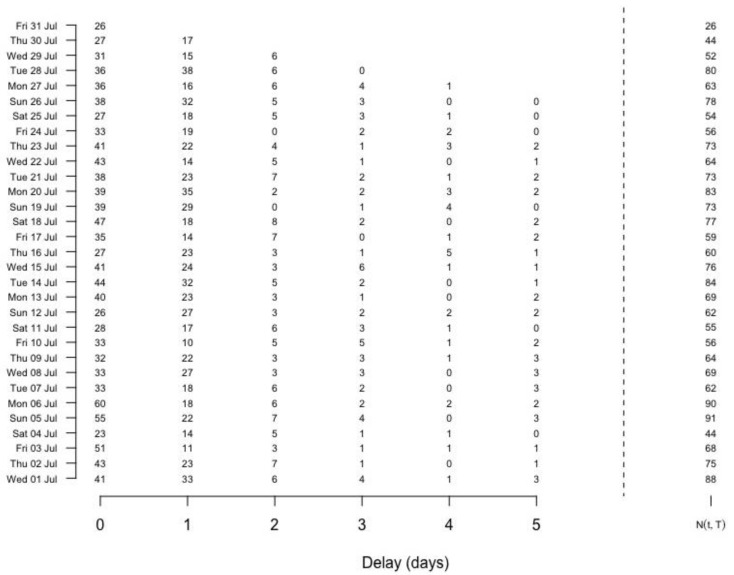
Reporting triangle at time 31 July 2015. Delays larger than 5 are covered in category “Delay = 5”.

By the delay distribution shown in Figure 4, we take the maximum delay D=5 days. Retrospectively, we can transform the historical available data into a final reporting triangle, which clearly shows the delay information. For example, in Figure 7, each cell $n_{t,d}$ indicates a count number that occurred at a given time point t but reported with a delay of d while the sum over all counts in a row is the corresponding $N_{t,T}$ for each day t shown on the right bars, where T= “31 July 2015”. Also we can work out the cumulative frequency for each delay based on the whole dataset, which provides informative prior for p.

### 4.2. Daily Surveillance

**Figure 8:**
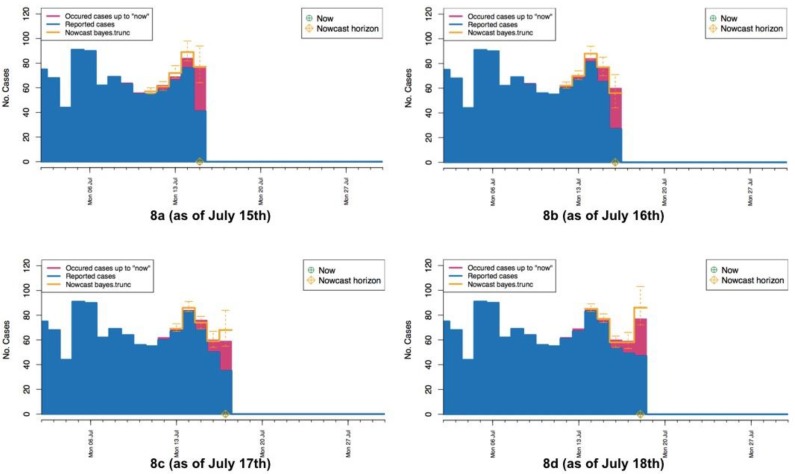
Pictures from animation of the nowcasting procedure (**a**–**d**) “now” = 15 July 2015–“now” = 18 July 2015. The yellow crosshairs indicate the current day *T*.

Below we apply the proposed BNT approach to the data set of foodborne patients described above. Here we set a time span 5 days to do the nowcasting. For each current day *T*, we try to predict the unobserved occurred cases $N_{t}$’s for t∈{T−4, T−3,T−2,T−1,T}. We give an animation of the details of our nowcasting process in the Supplementary Materials File S1. To explain the animation, we show four pictures for the continuous four days from 15 to 18 July in Figure 8. These pictures give the nowcasting process for predicting the numbers $N_{t}$’s of cases really occurred to the current days in July. Figure 8a takes 15 July as the current day, the numbers of reported cases until 15 July are drawn in blue, and the numbers of real occurred cases are drawn in red. Since there are 5 days for occurred cases to be reported, the accurate numbers of occurred cases for the current 5 days from 11 to 15 July may not be available as of 15 July. The goal is to predict the numbers of occurred cases for these current 5 days. In Figure 8a, the prediction of these numbers are drawn in yellow, the bold yellow bar indicates the median of the prediction distribution of occurred cases, the two short yellow bars indicate the 2.5% and 97.5% quantiles of the prediction distribution. The predicted number of occurred cases for 15 July is very close to the real number, although the interval between two bounds of the prediction distribution for 15 July is longer. The predicted number for 14 July is a little higher than the real number, although the interval is much shorter than that for 15 July. Similarly, Figure 8b–d show the predictions on 16–18 July, respectively, and it can be seen that interval for 15 July becomes shorter and shorter. From the four pictures drawn from the animation given in the Supplementary Materials File S1, we can see the predicted numbers are quite close to the real numbers of occurred cases for these four days, and the intervals all cover the real numbers of occurred cases.

### 4.3. Evaluating the Nowcasting

In this section, three different nowcasting procedures will be compared: the Lawless [18] frequency (LF) method with the consideration of the right-truncated nature and two Bayesian procedures, one is BNT method proposed in this paper and Bayesian nowcasting with no truncation (BNnT) method by Hohle and Heiden [20] ignoring the right truncation.

Before doing comparisons, we predict the numbers of occurred cases for 5 days on each current day *T*. We take *T* from 30 June 2015 to 30 August 2015, totally 63 days and so 5 × 63 = 315 nowcastings will be calculated. Considering a specific time T (for example “now”) we predict $N_{t}$’s for t∈{T−4, T−3,T−2,T−1,T}. To evaluate the performance of different nowcasting approaches, we use three scoring rules:

(i) Logarithmic score (logS) [26]:
  $$logS\left(P_{t}^{T},N_{t}\right)=-\log\left(f_{P_{t}^{T}}\left(N_{t}\right)\right)$$
(ii) Ranking probability score (RPS) [27,28]:
  $$RPS\left(P_{t}^{T},N_{t}\right)=\sum_{k}^{N}\left(F_{t}^{T}\left(N_{t}\right)-1\left(N_{t}\leq k\right)\right)$$
  where $P_{t}^{T}$ is the predictive distribution for time *t* based on the information available at *T* and with $N_{t}$ being the number of occurred cases. $f_{P_{t}^{T}}\left(\cdot \right)$ is the probability mass function (PMF) of the predictive distribution $P_{t}^{T}$, and where $F_{P_{t}^{T}}\left(\cdot \right)$ denotes the cumulative distribution function (CDF) of the predictive distribution $P_{t}^{T}$. And from the data prior information, here we choose $N_{max}=$ 300.
(iii) The proportion of times that the observed value lay outside the equal-tailed 95% predicted interval (OutCI).

Such rules allow investigating calibration and sharpness of predictive distribution. The higher probability of the forecast distribution for the actual observed value is, the better the prediction is. It means that the lower the score is, the better the performance is. Table 2 gives the mean scores obtained by averaging over the 315 nowcastings, which illustrates that BNT method proposed in this paper has an overall best performance based on these three evaluation rules compared to other existing methods (BNnT method and LF method).

**Figure 9:**
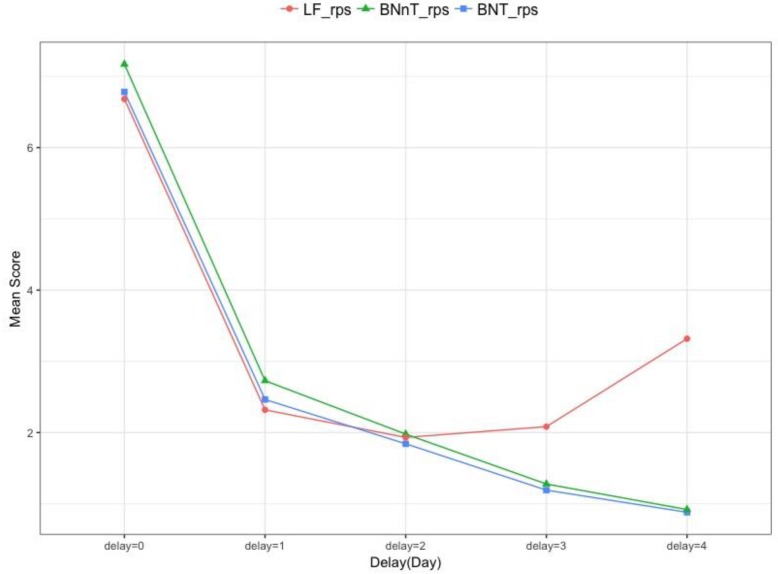
The mean scores of RPS comparison by different delays.

We consider the comparison among LF method, BNT method proposed in this paper and BNnT method, here we also predict the numbers of occurred cases for 5 days from the current day 0 to 4 days ago. Figure 9 shows the mean scores of RPS obtained by averaging for t∈{T−4, T−3,T−2,T−1,T} from 315 nowcastings. The mean scores are the largest for delay=0 among all delays. When the delay increases, more information is collected, and the LF method (red line) and BNT method (blue line) have a better performance with delay≤2. When delay≥2 days, the BNT method appears to perform best.

**Figure 10:**
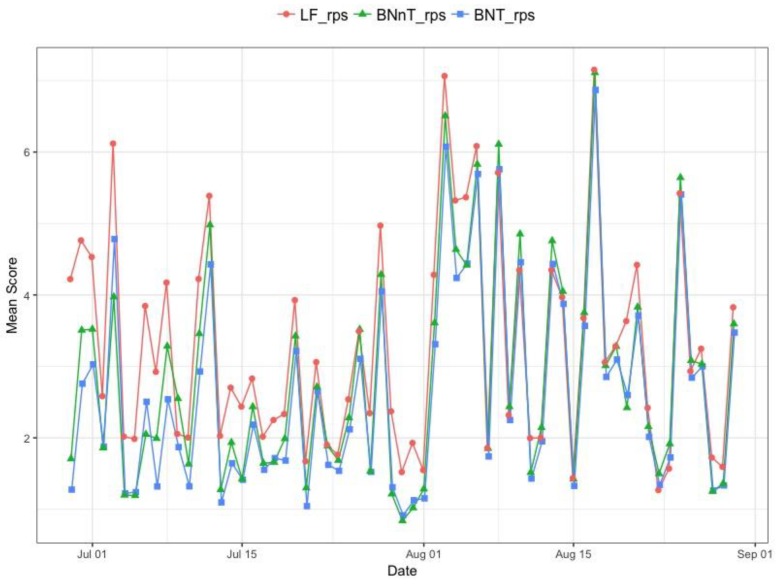
The Mean score as a function of the time points.

Finally, we compare the mean scores of RPS among LF method, BNT method proposed in this paper and BNnT method with considering the time span from 1 July to 30 August. Figure 10 shows that BNT method (blue line) outperforms the LF method (red line) and BNnT method (green line).

## 5. Discussion

In this paper, we introduced a Bayesian hierarchal approach to monitor and forecast in real-time Chinese foodborne disease outbreaks based on the public health surveillance data if reporting delays are present. Such delay-adjusting tracking procedures can provide daily information on the epidemic trends by predicting the daily true number of patients.

In fact, the delay could be divided into two phases. Phase 1 is the delay from the onset to the doctor visit, and this is the delay of the patients in seeking medical treatments. Since the foodborne disease is not a serious disease requiring immediate emergency, many patients are not in a hurry to go to the hospital for treatment, which may cover up the outbreak of a food safety incident. Phase 2 is the delay between the visit date and the hospital reporting date. The delay distribution in this stage is very complicated. Due to the difference for medical level of each hospital, the efficiency of the experiment, the doctor’s understanding of the foodborne disease and the definition of the cases, etc., the diagnosis report will be delayed. This is the delay in phase 2. References [19,20,21] used the number of patients reported in the hospital to predict the number of occurred cases. The cycle is very long from the onset to the diagnosis reported, which couldn’t predict the number of cases, or grasp the trends of the disease in a timely way. Therefore, in view of this, our model has the following advantages: First, when the data is complete and there is hospital-confirmed report data in hand, our model uses the number of confirmed patients to predict the number of occurred cases, a procedure similar to that of [20,21]. Then, if the data is incomplete, for example, we only have the visiting data till today, we could use the number of visiting patients to predict the occurred cases, which may lead to a very small overestimation of the number of occurred cases, but least not underestimate them. However, it makes the prediction ahead of days (the delay in Phase 2). In fact, our collected data shows that there are 21,226 confirmed cases among 21,866 visiting patients, a proportion as high as 97%. Early warning of disease outbreaks could avoid long delays from patient visit to disease confirmation, which can greatly shorten the forecast period, detecting possible food safety incidents in a more timely fashion. It is very significant in this regard.

As future work, since the number of visits is used to predict the number of occurred cases that will be overestimated (at least not underestimated), we will use the empirical data to obtain the proportion of confirmed cases among the visiting patients. Under this condition, the information of the proportion can be used to predict the number of occurred cases. We will also consider applying a compound Poisson model to solve the problem of overestimation of the number of occurred cases. Meanwhile, other covariates including city, age, occupation, etc., will also be considered. How to analyze and model the foodborne disease surveillance data, and how to detect the outbreak based on historical data, even the casual inference to pathogenic factors, are all issues to be studied in the future. 

## Figures and Tables

**Table 1:** Example of the information collected for the Data set.

Patient ID	Hospital Information			Onset Date	Visit Date	Confirmed
	Province	City	Sentinel Hospital			
HN073408-2015-00040	Hunan	Changsha	The Fourth Hospital of Changsha	10 September 2015	11 September 2015	Yes
HN073402-2015-00086	Hunan	Hengyang	Hengyang Centre Hospital of Hunan	07 June 2015	10 June 2015	Yes
HN073002-2015-00128	Hunan	Yueyang	Yueyang Second People’s Hospital	30 September 2015	30 September 2015	Yes

**Table 2:** Mean scores for different nowcasting methods.

Method	RPS	logS	OutCI
BNT	2.63	2.52	0.07
BNnT	2.81	2.80	0.12
LF	3.27	2.58	0.07

## Supplementary Materials

The following are available online at http://www.mdpi.com/1660-4601/15/8/1740/s1, File S1. Nowcast Animation. This PDF file contains an animation of available reports as a function of time (in days) in the period 01 July 2015 to 30 July 2015.
