# Addendum: Wang et al. A Bayesian Approach to Real-Time Monitoring and Forecasting of Chinese Foodborne Diseases. *Int. J. Environ. Res. Public Health*, 2018, 15(8):1740; doi:10.3390/ijerph15081740

**DOI:** 10.3390/ijerph16081442

**Published:** 2019-04-23

**Authors:** Xueli Wang, Moqin Zhou, Jinzhu Jia, Zhi Geng, Gexin Xiao

**Affiliations:** 1 School of Science, Beijing University of Posts and Telecommunications, Beijing 100876, China; wangxl@bupt.edu.cn (X.W.); interpreter_q@hotmail.com (M.Z.); 2 School of Public Health, Center of Statistical Science, Peking University, Beijing 100871, China; jzjia@math.pku.edu.cn; 3 School of Mathematical Sciences, Center of Statistical Science, Peking University, Beijing 100871, China; zhigeng@pku.edu.cn; 4 China National Center for Food Safety Risk Assessment, Beijing 100022, China

The authors wish to update the Abstract and Section 3 in their paper published in the *International Journal of Environmental Research and Public Health (IJERPH)* [1].

They would like to rewrite the abstract as follows:

**Abstract:** Foodborne diseases have a big impact on public health and are often underreported. This is because a lot of patients delay treatment when they suffer from foodborne diseases. In Hunan Province (China), a total of 21,226 confirmed foodborne disease cases were reported from 1 March 2015 to 28 February 2016 by the Foodborne Surveillance Database (FSD) of the China National Centre for Food Safety Risk Assessment (CFSA). The purpose of this study was to make use of the daily number of visiting patients to forecast the daily true number of patients. Our main contribution is that we take the reporting delays into consideration and apply a Bayesian hierarchical model for this forecast problem. The data shows that there were 21,226 confirmed cases reported among 21,866 visiting patients, a proportion as high as 97%. Given this observation, the Bayesian hierarchical model was established to predict the daily true number of patients using the number of visiting patients. We use several scoring rules to assess the performance of different nowcasting procedures. We conclude that Bayesian nowcasting with consideration of right truncation of the reporting delays has a good performance for short-term forecasting and could effectively predict the epidemic trends of foodborne diseases. Meanwhile, this approach could provide a methodological basis for future foodborne disease monitoring and control strategies, which are crucial for public health. 

In the end of the first paragraph in Section 3, the authors would like to update the last two sentences of the paragraph and add some citations. The revised sentences are as follows:

In this paper, we apply a Bayesian nowcasting model proposed by Höhle and an der Heiden [2] to forecast the daily total number of cases. Thanks to Salmon et al. [3], who provided a convenient R package “surveillance”, the inference for the model could be easily performed. The R package surveillance also contains a few other nowcasting methods that we also tried and did comparisons with using the scoring rules implemented in the package. The results are shown in Section 4. Below we review the model.

The authors revised Section 3.2 to describe the inference approach proposed by Höhle and an der Heiden (in Section 3.2 of [2]) in some greater detail. This part is now as follows:

For the convenience of the reader, we describe the inference approach proposed by Höhle and an der Heiden (in Section 3.2 of [2]) in some greater detail.

Define $p_{d}$ as the (time-homogeneous) probability that a case will have a reporting delay of d days. The $p_{d}$’s satisfy the following equation: $\sum_{d=0}^{D}p_{d}=1$. Following Kalbfleisch and Lawless’s previous work [4] and Zeger et al.’s previous work [5], we assume that the occurrence time of cases follows an underlying inhomogeneous Poisson process. A reasonable data generating process for the daily number of cases is thus as follows:

$$\begin{array}{cc} N_{t}|\lambda_{t} & ∼\mathrm{Po}\left(\lambda_{t}\right)\\ \left(n_{t,D},n_{t,D-1},\cdots ,n_{t,0}\right)\prime |N_{t},p & ∼\mathrm{MN}\left(N_{t},\left(p_{D},p_{D-1},\cdots ,p_{0}\right)\prime \right) \end{array}$$

where Po(λ) denotes the Poisson distribution with expectation λ>0 and MN(N,p) denotes the multinomial distribution with size parameter N and probability vector p. Nowcasting for a given time T can thus be divided into steps of determining the $\lambda_{t}$’s, estimating the unknown delay distribution (i.e., the $p_{d}$’s), and finally predicting the unobserved $n_{t,d}$’s in order to compute the total $N_{t}$. As T increases, and if the assumption about a time-homogeneous delay distribution is acceptable, the available data make it possible to estimate the delay distribution better and better, and hence the quality of the predictions near T improves with time.

Consider a fixed time T and define $p_{T}=\left(p_{T,D},p_{T,D-1},\cdots ,p_{T,1}\right)\prime$ as the probability vector denoting that a case is reported with a delay of d days given the observed incomplete information at time T, i.e., the set of $n_{t,d},$ where t+d≤T. We choose as prior distribution the generalised Dirichlet distribution GD(α,β) with fixed constants $\alpha =\left(\alpha_{0},\alpha_{1},\cdots ,\alpha_{D}\right)\prime$ and $\beta =\left(\beta_{0},\beta_{1},\cdots ,\beta_{D}\right)\prime$. Now we use Property 3 in the Web appendix of [2] that shows that the posterior of p under right-truncated multinomial sampling is again a GD distribution with parameters $\alpha_{T}^{*},\beta_{T}^{*}$ given by

$$\begin{array}{ll} \alpha_{T,i}^{*} & =\alpha_{i}+\overset{T-D+i}{\underset{\tau =0}{\sum }}n_{\tau ,D-i}\\ \beta_{T,i}^{*} & =\beta_{i}+\overset{T-D+i}{\underset{\tau =0}{\sum }}(N_{\tau ,\tau +D-i}-n_{\tau ,D-i}),\;i\in \left\{0,\cdots ,D-1\right\}. \end{array}$$

hence, for a given T we can assume the following model hierarchy for the time points t∈{T−D,⋯,T}:

$$\begin{array}{ll} p_{T} & ∼GD\left(\alpha_{T}^{*},\beta_{T}^{*}\right)\\ \lambda_{t} & ∼Ga\left(a_{\lambda },b_{\lambda }\right)\\ N_{t}|\lambda_{t} & ∼Po\left(\lambda_{t}\right)\\ N_{t,T}|N_{t},p_{T} & ∼\mathrm{Bin}\left(N_{t},q_{T,T-t}\right), \end{array}$$

where $q_{T,d}=\sum_{\delta =0}^{d}p_{T,\delta }$ is the proportion reported within a delay of d days. We denote by $Ga\left(a_{\lambda },b_{\lambda }\right)$ the gamma distribution with parameters $a_{\lambda }>0,b_{\lambda }>0$. For this hierarchical model, the marginal distribution of $N_{t}$ is a negative binomial distribution with the following mean and variance:

$$\begin{array}{cc} E\left(N_{t}\right) & =\mu_{\lambda }=a_{\lambda }b_{\lambda }\\ \mathrm{Var}\left(N_{t}\right) & =\mu_{\lambda }+\frac{\mu_{\lambda }^{2}}{a_{\lambda }}. \end{array}$$

To estimate $N_{t}$ given the observed counts $n_{t,d}$ at time T, we have to perform two steps: (1) update the delay distribution $q_{T}$ and (2) update the prediction for $N_{t}$:

(1) For the given T we compute $\alpha_{T}^{*},\beta_{T}^{*}$ as stated above. We then draw for k=1,…,K random vectors $p_{T}^{\left(k\right)}∼GD\left(\alpha^{*}{}_{T},\beta_{T}^{*}\right)$ by the algorithm of Wong [6] and calculate
  $$q_{T,d}^{\left(k\right)}=\overset{d}{\underset{\delta =0}{\sum }}p_{T,\delta }^{\left(k\right)}.$$
(2) Given the updated delay distribution $q_{T}^{\left(k\right)}$ and the observed counts $n_{t,d}$, we can now update the prediction of $N_{t},t=T-D,\cdots ,T$.
  For n∈{0,1,2,⋯} we approximate by Monte Carlo sampling
  $$f(N_{t}=n|N_{t,T})\approx \frac{1}{K}\overset{K}{\underset{k=1}{\sum }}f_{n,t}^{\left(k\right)},\;t\in \left\{T-D,\cdots ,T\right\}$$
  An application of Bayes theorem provides $f_{n,t}^{\left(k\right)}={\tilde{f}}_{n,t}^{\left(k\right)}/c_{t}^{\left(k\right)}$, where $c_{t}^{\left(k\right)}=\sum_{n=0}^{\infty }{\tilde{f}}_{n,t}^{\left(k\right)}$ is the normalization constant and
  $${\tilde{f}}_{n,t}^{\left(k\right)}=f(N_{t,T}|N_{t}=n,q_{T,T-t}^{\left(k\right)})f(N_{t}=n|\lambda_{t})f\left(\lambda_{t}\right)$$
  for all t∈{T−D,⋯,T}. The factors of the last equation can be evaluated using the distributional assumptions of the model hierarchy. For numerical convenience we do not sum over the entire support {0,1,2,⋯} to get the normalization, but instead approximate
  $$c_{t}^{\left(k\right)}\approx \overset{N_{\max}}{\underset{n=0}{\sum }}{\tilde{f}}_{n,t}^{\left(k\right)},$$
  where $N_{\max}$ is chosen sufficiently large.

Finally, the authors would like to update “proposed in this paper” to “described in this paper”.

The changes do not affect the results. The manuscript will be updated and the original will remain online on the article webpage, with a reference to this addendum.
