# Supplementary material for: A Bayesian Approach to Real-Time Monitoring and Forecasting of Chinese Foodborne Diseases
Source: Int J Environ Res Public Health. 2018 Aug 13;15(8):1740. doi: 10.3390/ijerph15081740 (PMC6121893; doi:10.3390/ijerph15081740)
Supplement: Supplementary file 1 [file ijerph-15-01740-s001.zip › S1 NowcastAnimation.pdf]

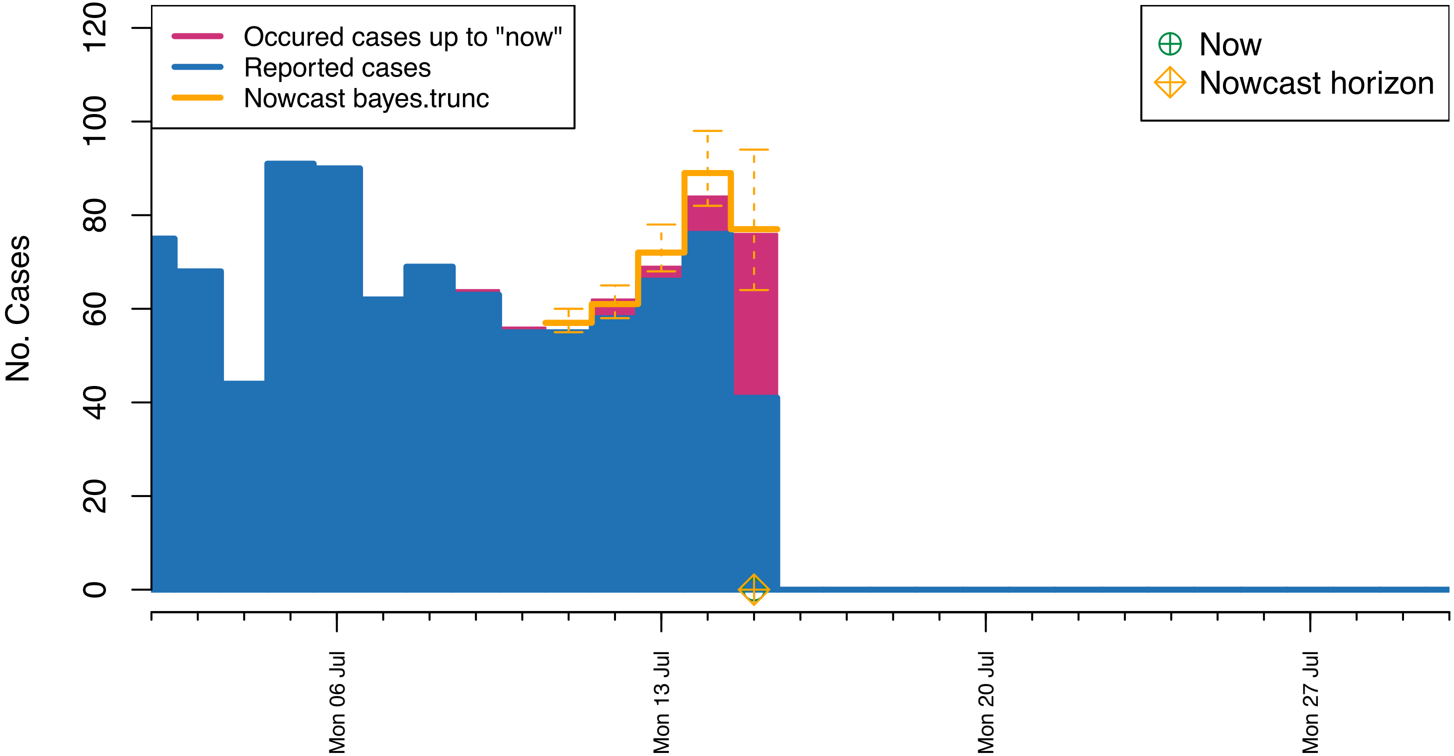

Occured cases up to "now"
Reported cases
Nowcast bayes.trunc
Now
Nowcast horizon
No. Cases
0
20
40
60
80
100
120
Mon 06 Jul
Mon 13 Jul
Mon 20 Jul
Mon 27 Jul

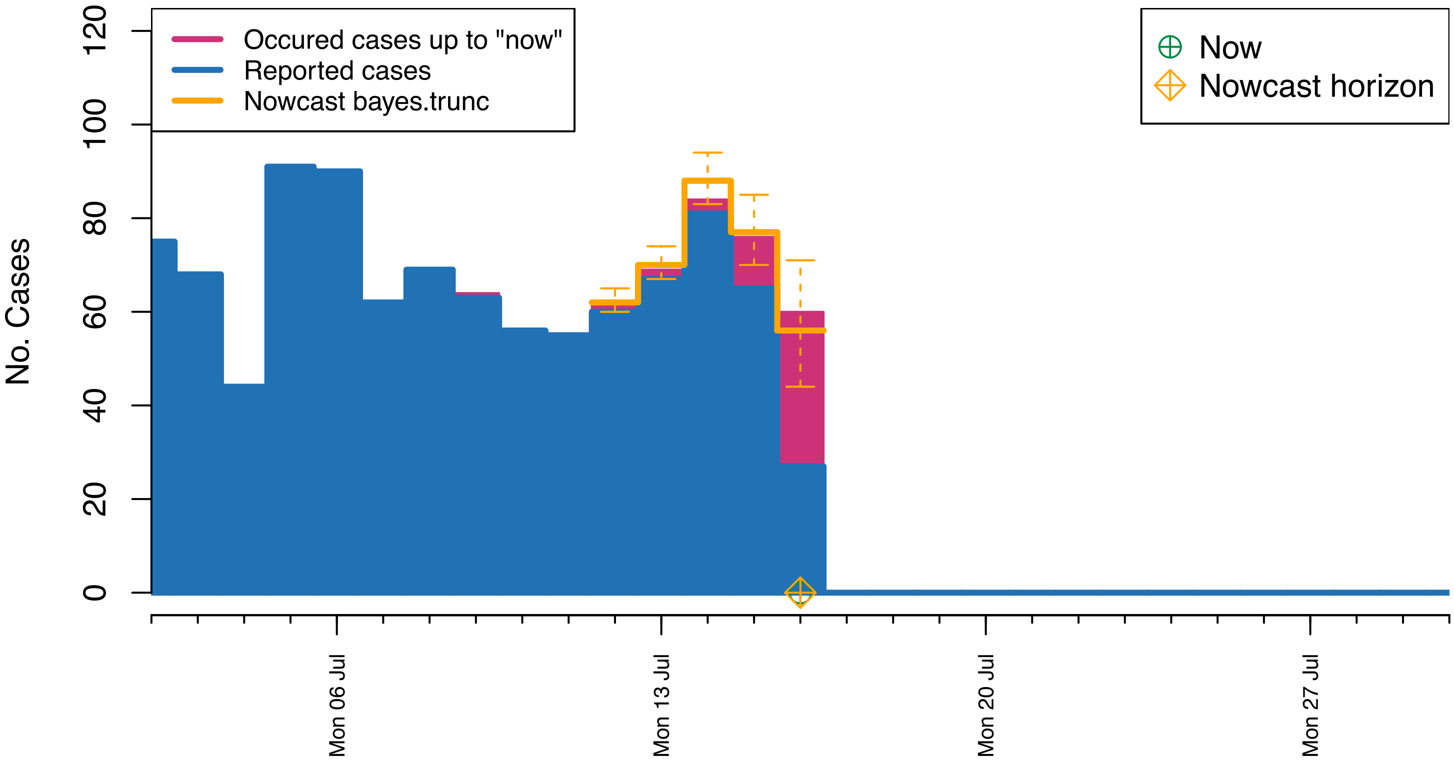

Occured cases up to "now"
Reported cases
Nowcast bayes.trunc
Now
Nowcast horizon
No. Cases
0
20
40
60
80
100
120
Mon 06 Jul
Mon 13 Jul
Mon 20 Jul
Mon 27 Jul

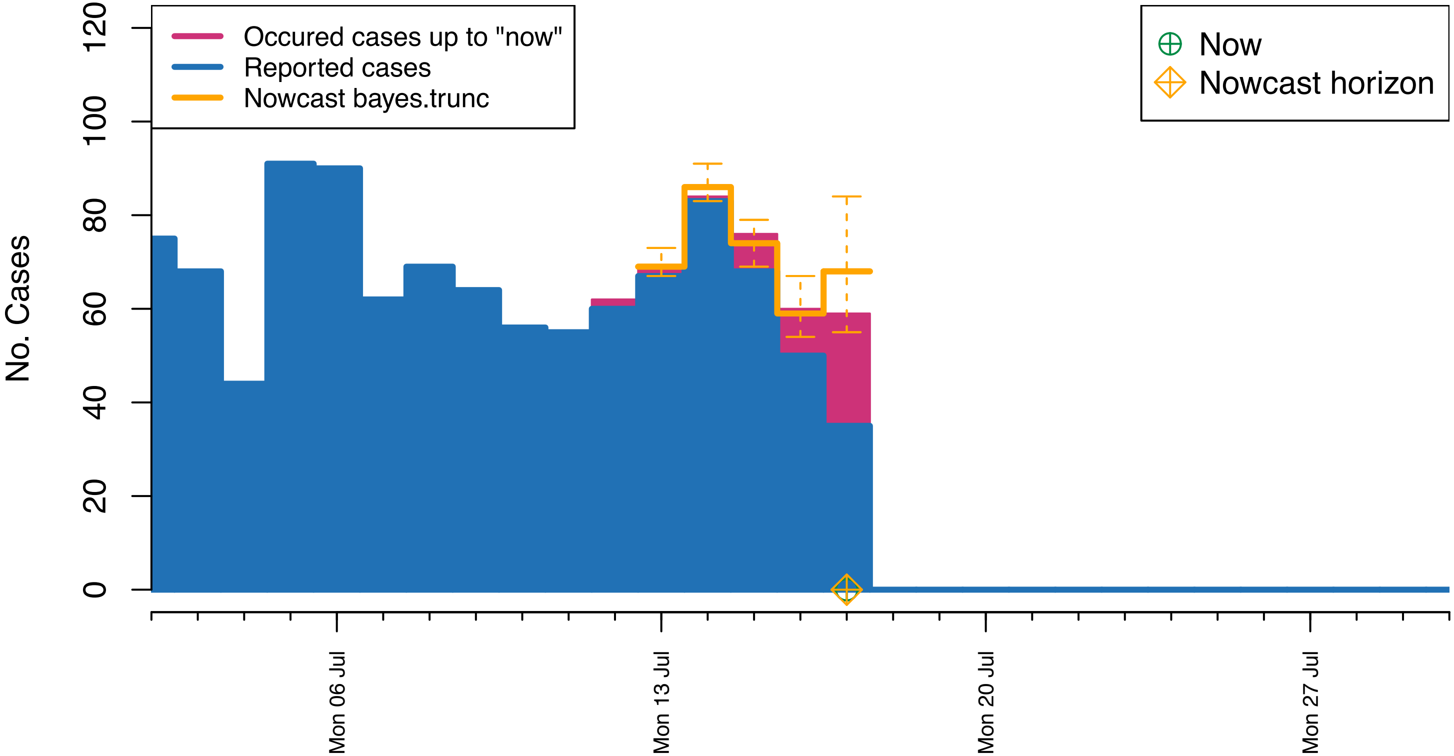

Occured cases up to "now"
Reported cases
Nowcast bayes.trunc
Now
Nowcast horizon
No. Cases
0
20
40
60
80
100
120
Mon 06 Jul
Mon 13 Jul
Mon 20 Jul
Mon 27 Jul

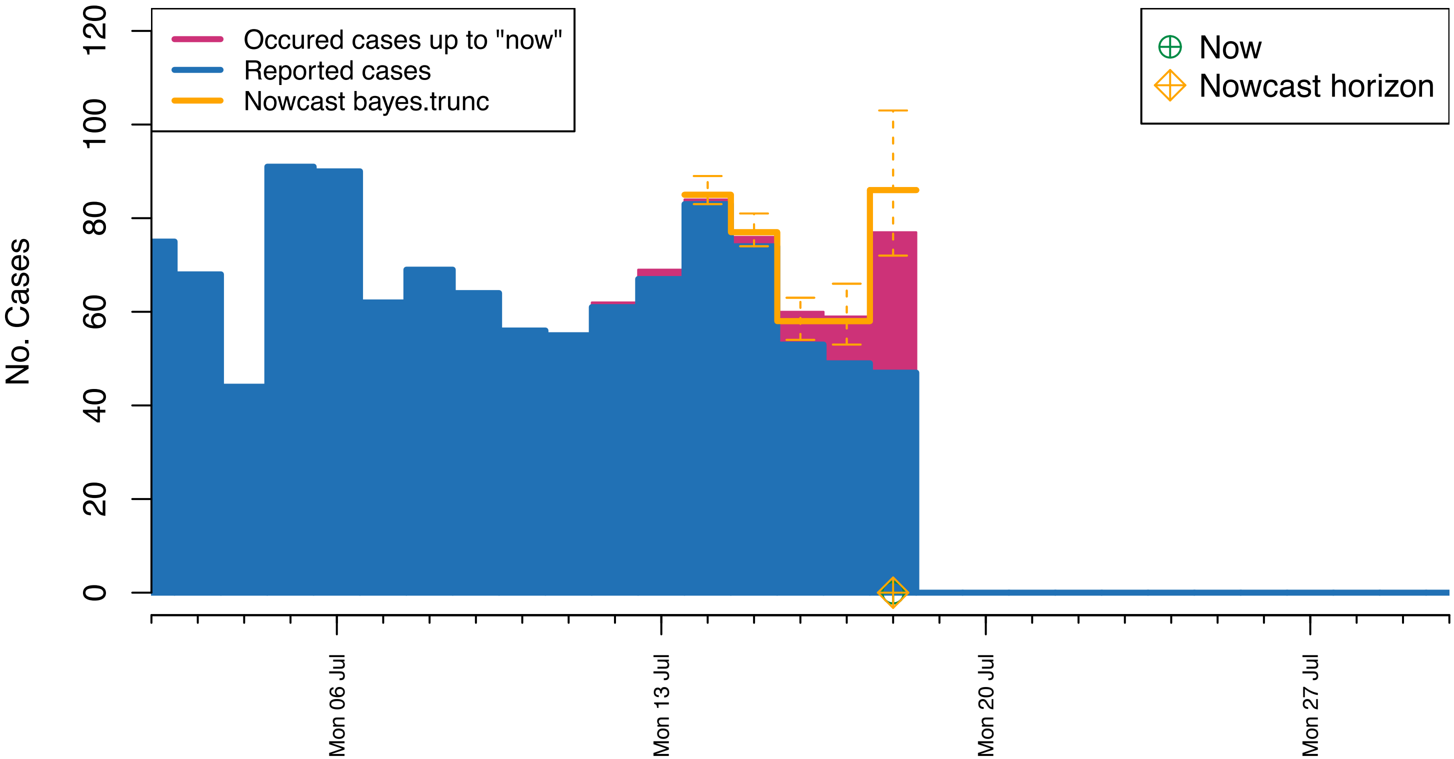
Occured cases up to "now"
Reported cases
Nowcast bayes.trunc
Now
Nowcast horizon
No. Cases
0
20
40
60
80
100
120
Mon 06 Jul
Mon 13 Jul
Mon 20 Jul
Mon 27 Jul

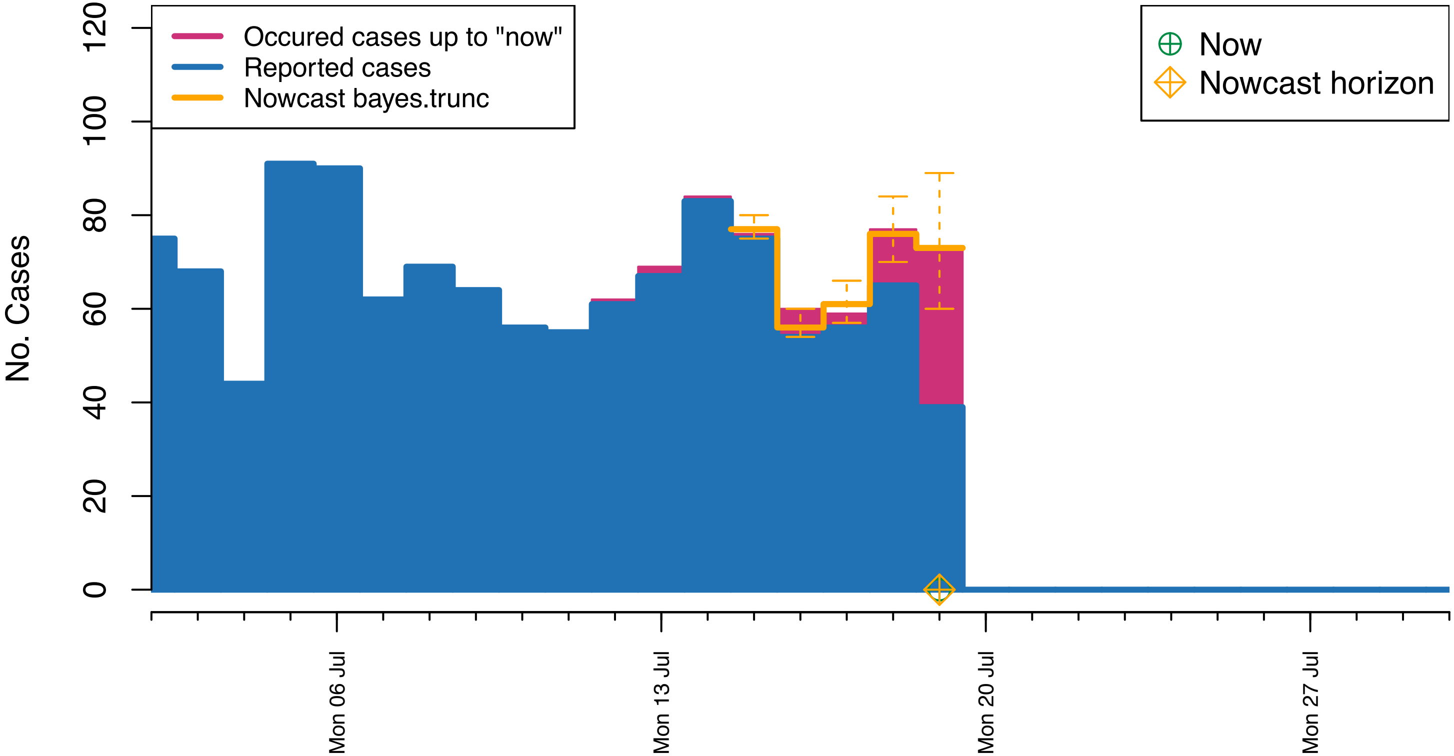

Occured cases up to "now"
Reported cases
Nowcast bayes.trunc
Now
Nowcast horizon
No. Cases
0
20
40
60
80
100
120
Mon 06 Jul
Mon 13 Jul
Mon 20 Jul
Mon 27 Jul

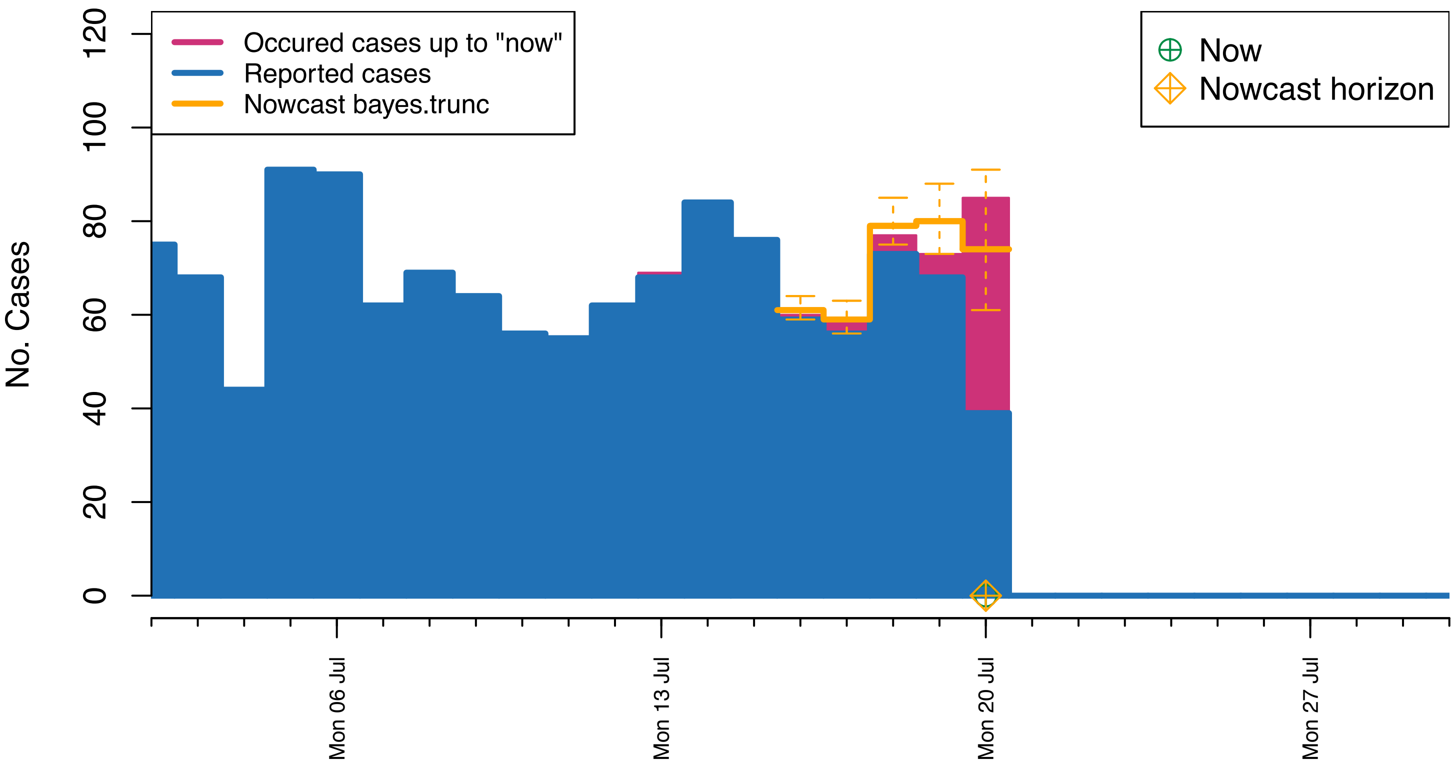

Occured cases up to "now"
Reported cases
Nowcast bayes.trunc
Now
Nowcast horizon
No. Cases
0
20
40
60
80
100
120
Mon 06 Jul
Mon 13 Jul
Mon 20 Jul
Mon 27 Jul

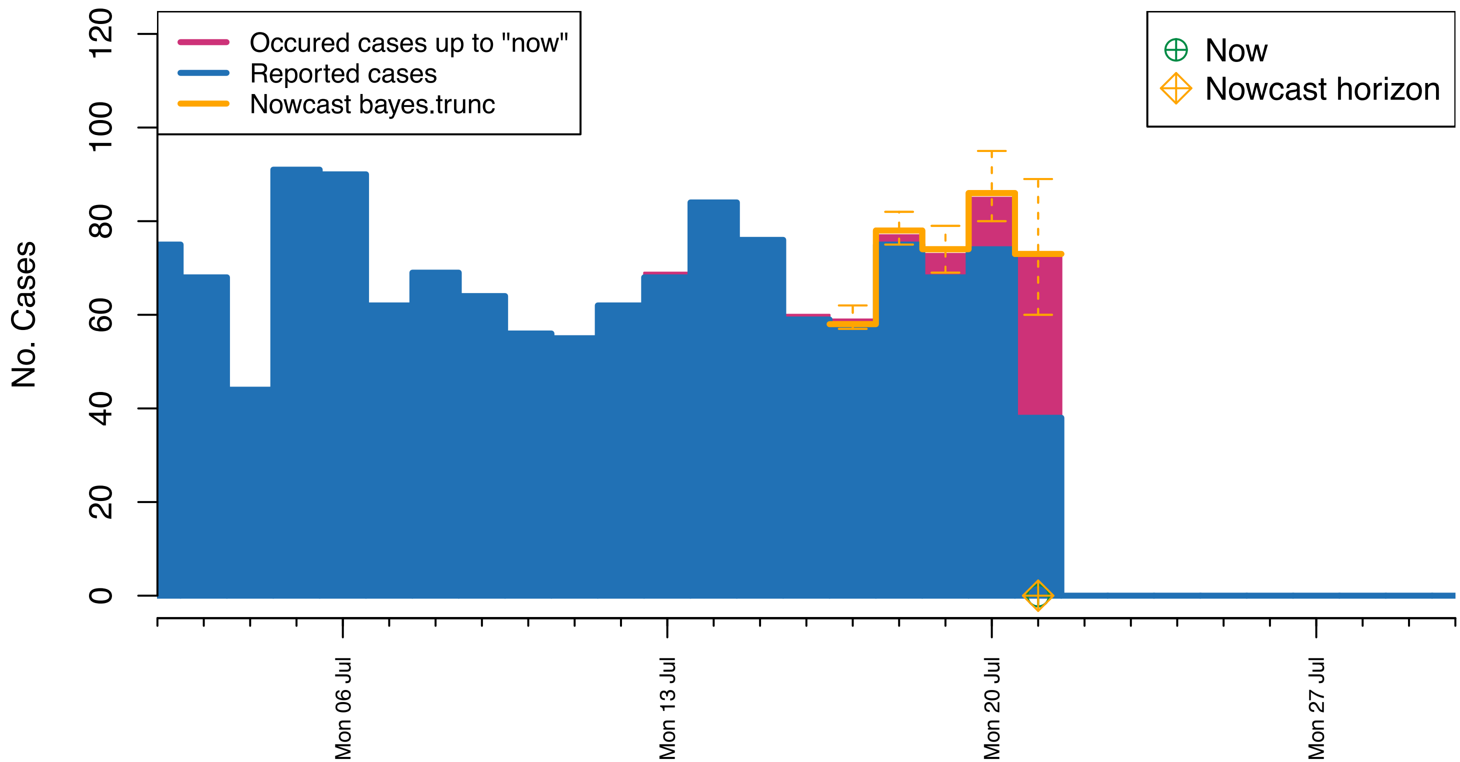

Occured cases up to "now"
Reported cases
Nowcast bayes.trunc
Now
Nowcast horizon
No. Cases
0
20
40
60
80
100
120
Mon 06 Jul
Mon 13 Jul
Mon 20 Jul
Mon 27 Jul

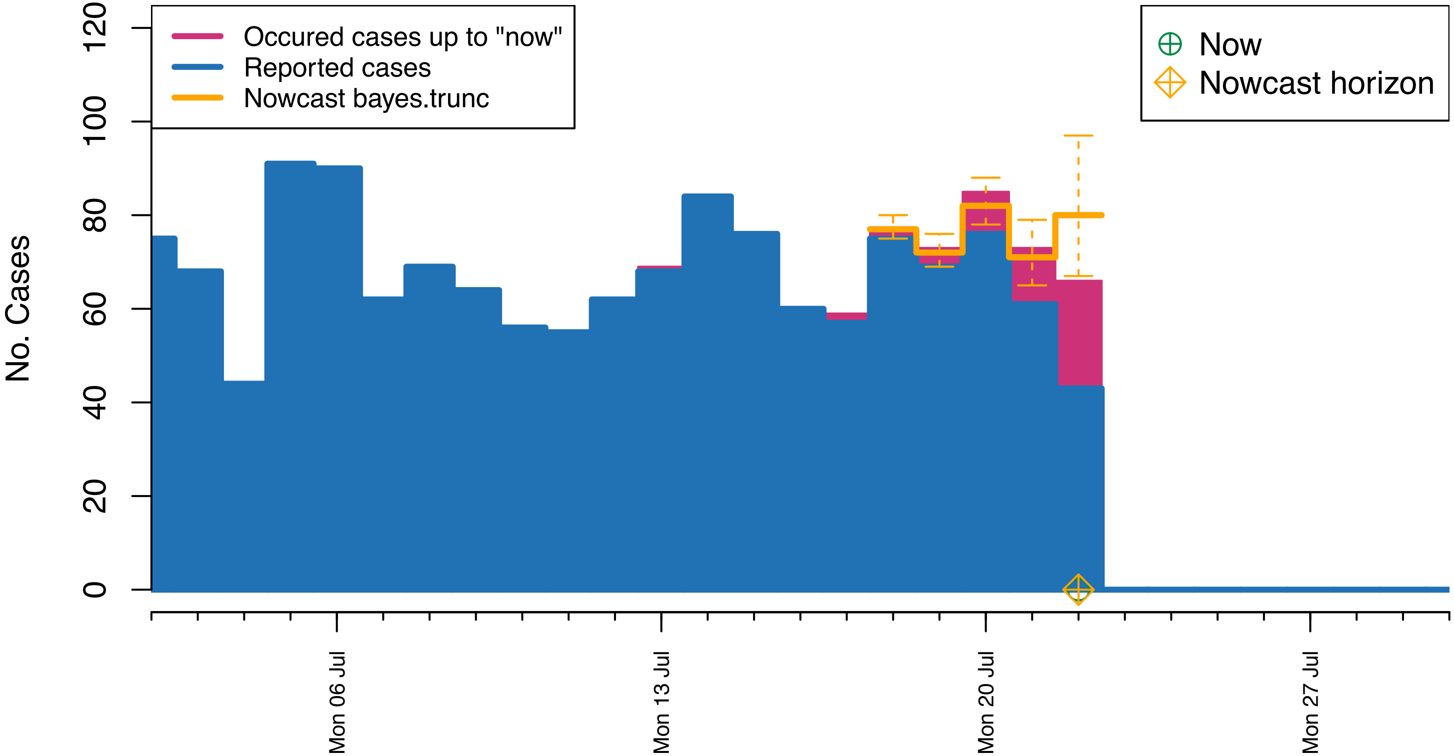

Occured cases up to "now"
Reported cases
Nowcast bayes.trunc
Now
Nowcast horizon
No. Cases
0
20
40
60
80
100
120
Mon 06 Jul
Mon 13 Jul
Mon 20 Jul
Mon 27 Jul

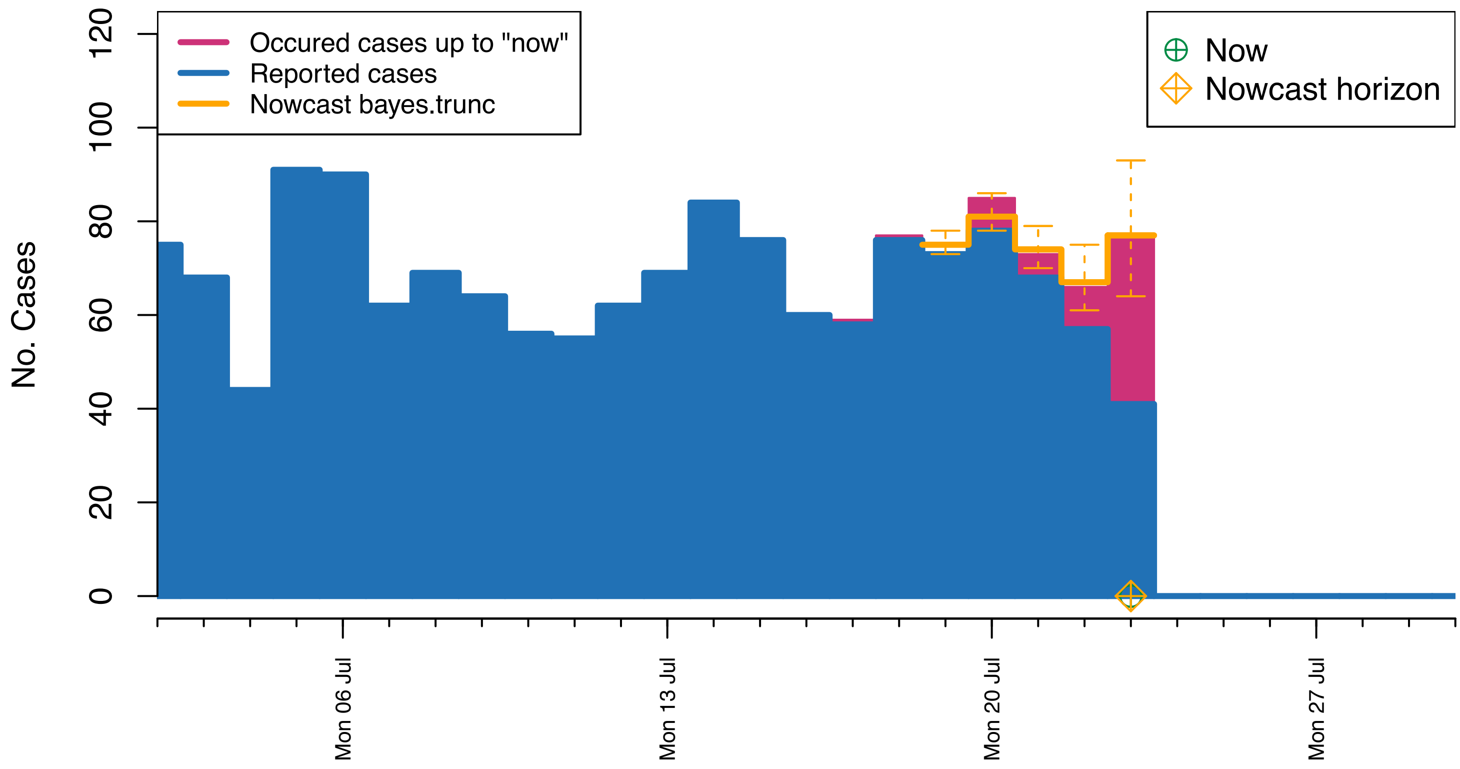
Occured cases up to "now"
Reported cases
Nowcast bayes.trunc
Now
Nowcast horizon
No. Cases
0
20
40
60
80
100
120
Mon 06 Jul
Mon 13 Jul
Mon 20 Jul
Mon 27 Jul

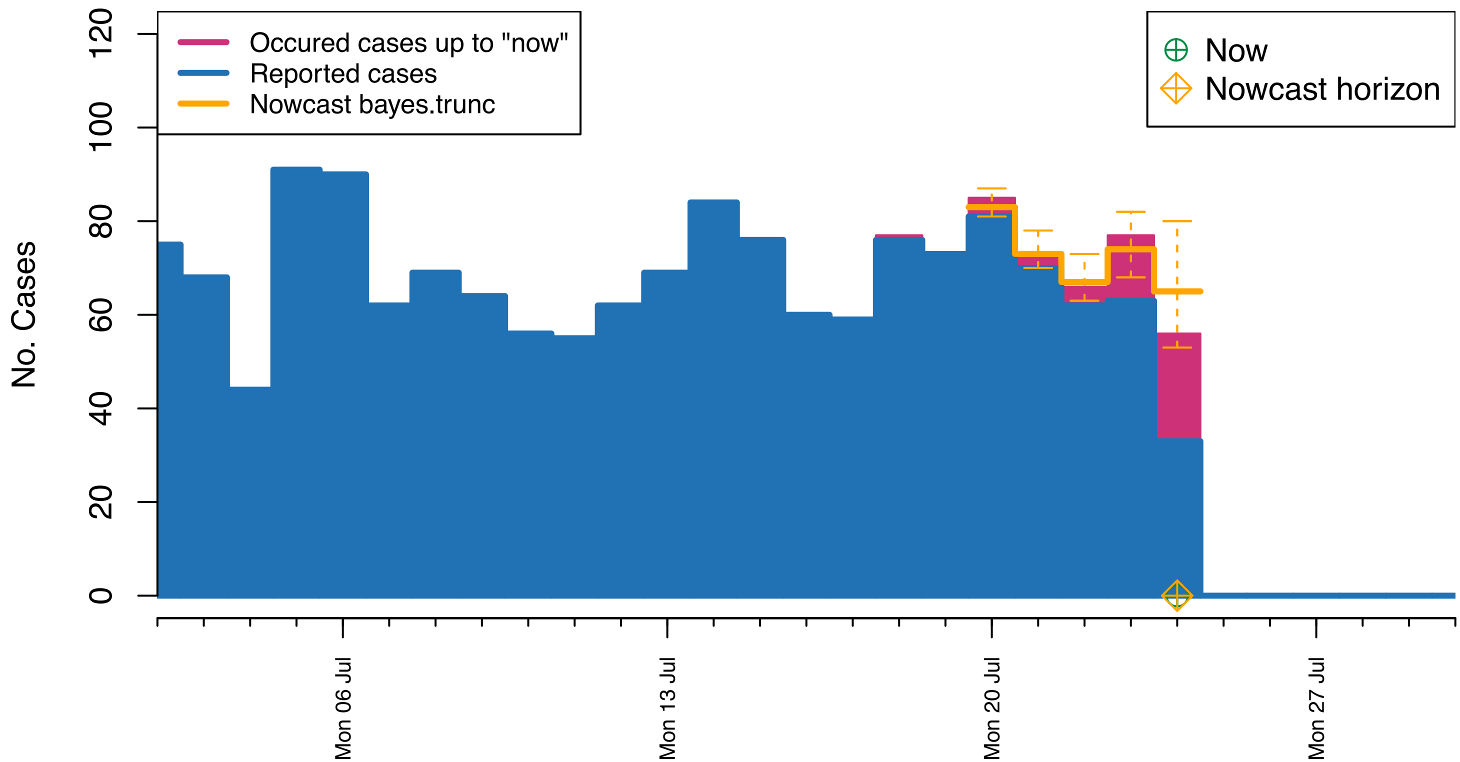

Occured cases up to "now"
Reported cases
Nowcast bayes.trunc
Now
Nowcast horizon
No. Cases
0
20
40
60
80
100
120
Mon 06 Jul
Mon 13 Jul
Mon 20 Jul
Mon 27 Jul

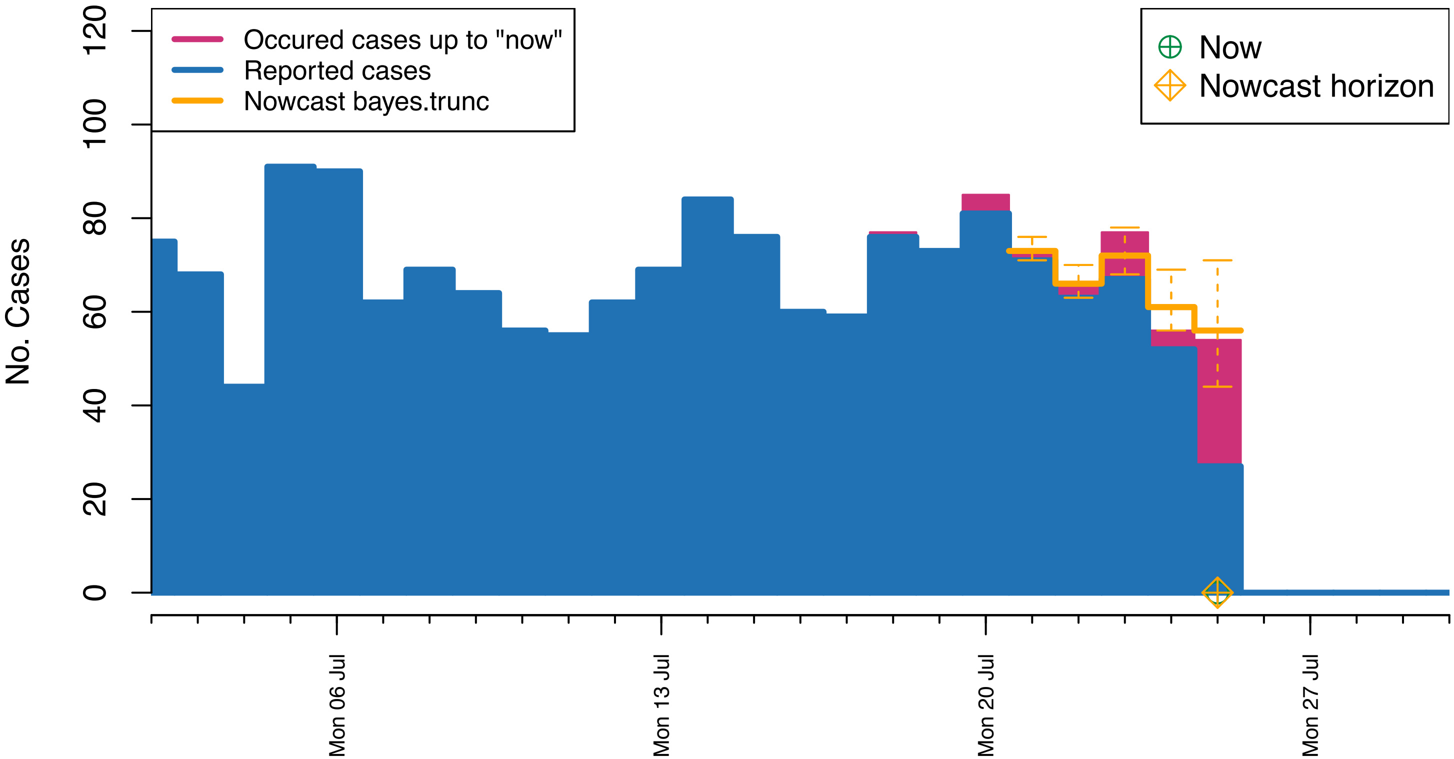

Occured cases up to "now"
Reported cases
Nowcast bayes.trunc
Now
Nowcast horizon
No. Cases
0
20
40
60
80
100
120
Mon 06 Jul
Mon 13 Jul
Mon 20 Jul
Mon 27 Jul

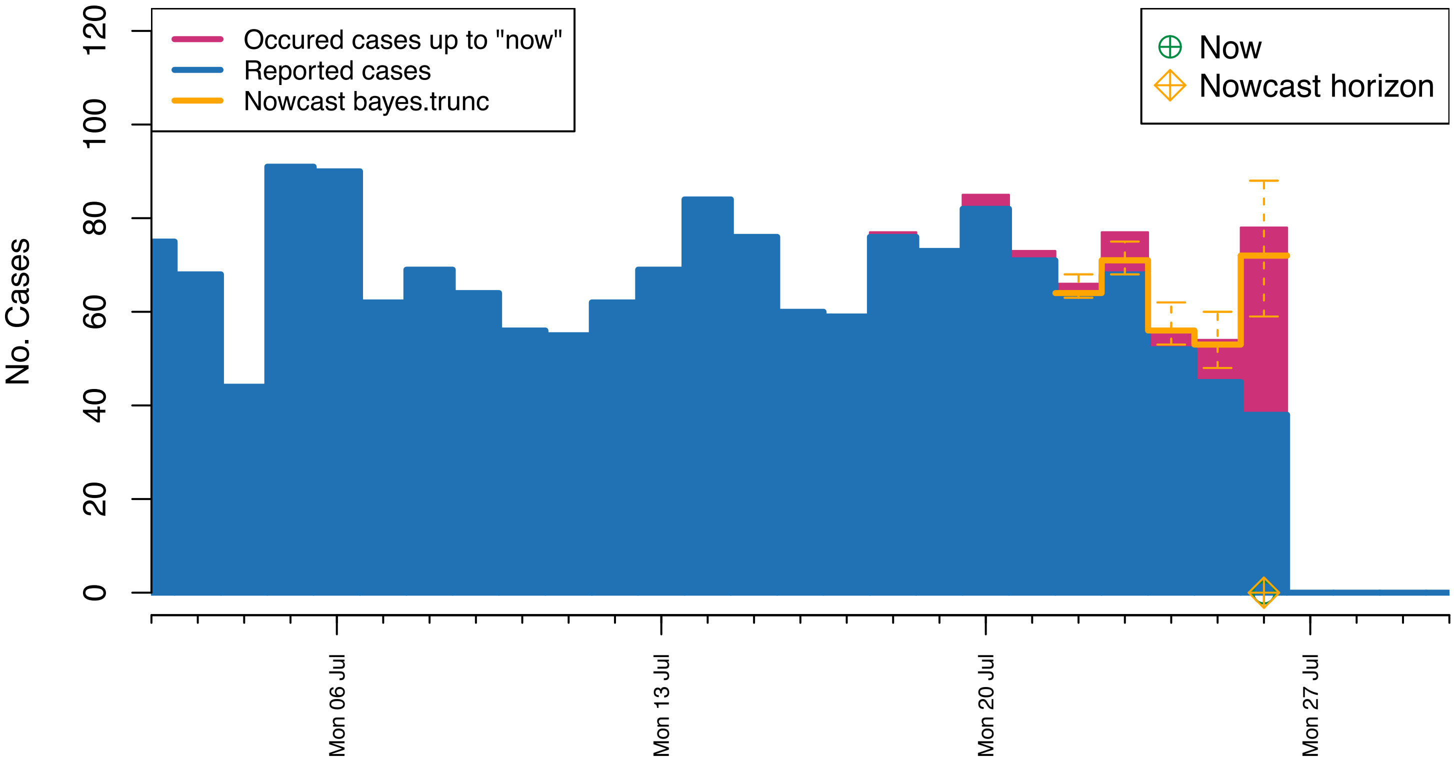

Occured cases up to "now"
Reported cases
Nowcast bayes.trunc
Now
Nowcast horizon
No. Cases
0
20
40
60
80
100
120
Mon 06 Jul
Mon 13 Jul
Mon 20 Jul
Mon 27 Jul

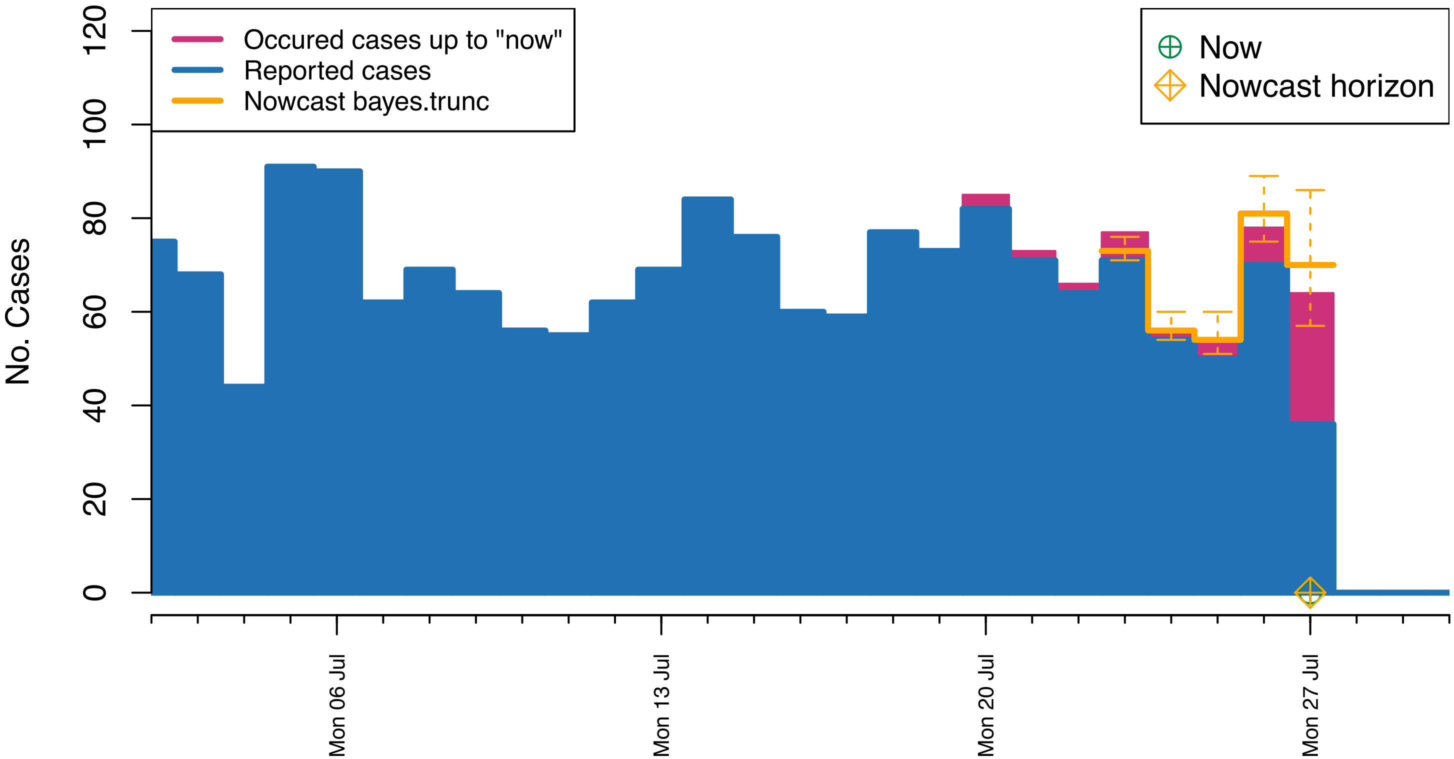
Occured cases up to "now"
Reported cases
Nowcast bayes.trunc
Now
Nowcast horizon
No. Cases
0
20
40
60
80
100
120
Mon 06 Jul
Mon 13 Jul
Mon 20 Jul
Mon 27 Jul

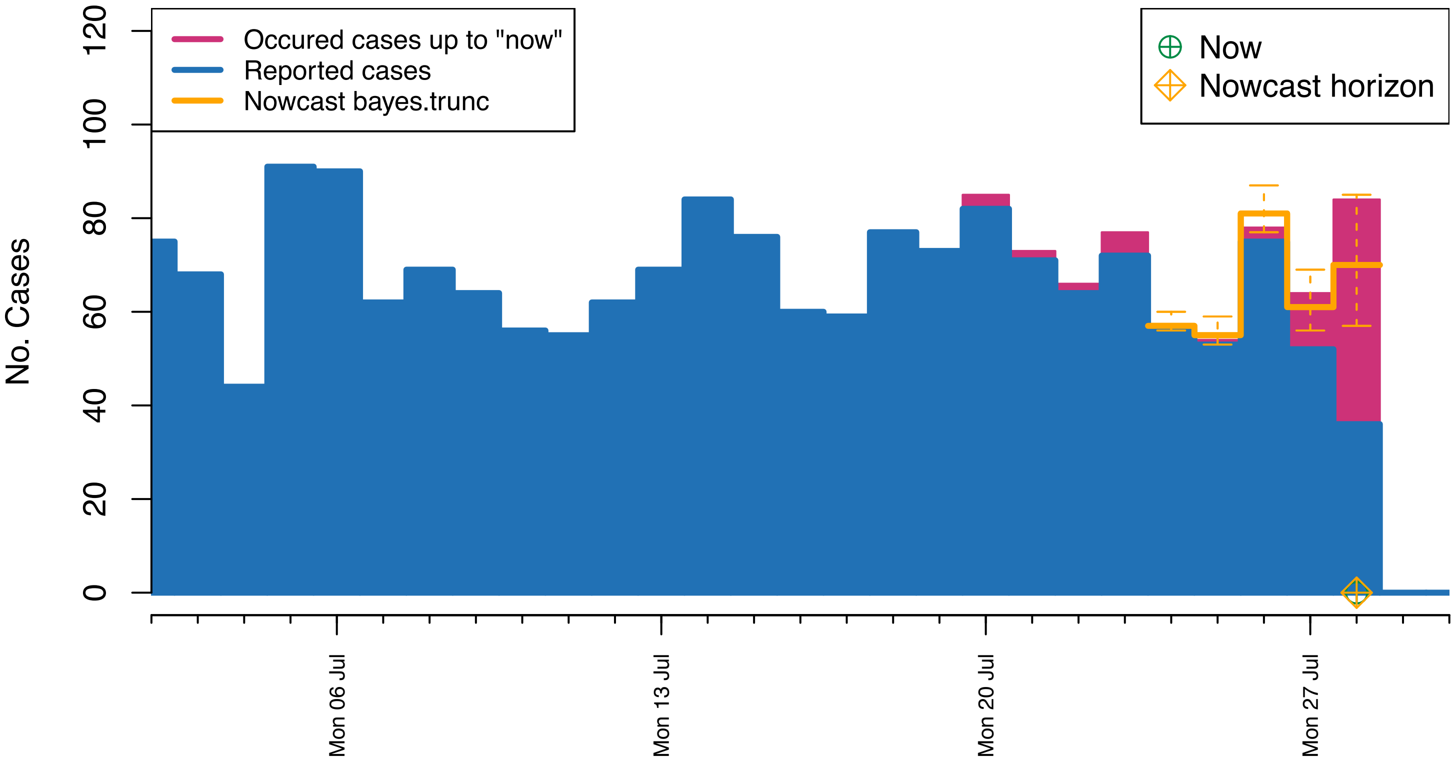

Occured cases up to "now"
Reported cases
Nowcast bayes.trunc
Now
Nowcast horizon
No. Cases
0
20
40
60
80
100
120
Mon 06 Jul
Mon 13 Jul
Mon 20 Jul
Mon 27 Jul

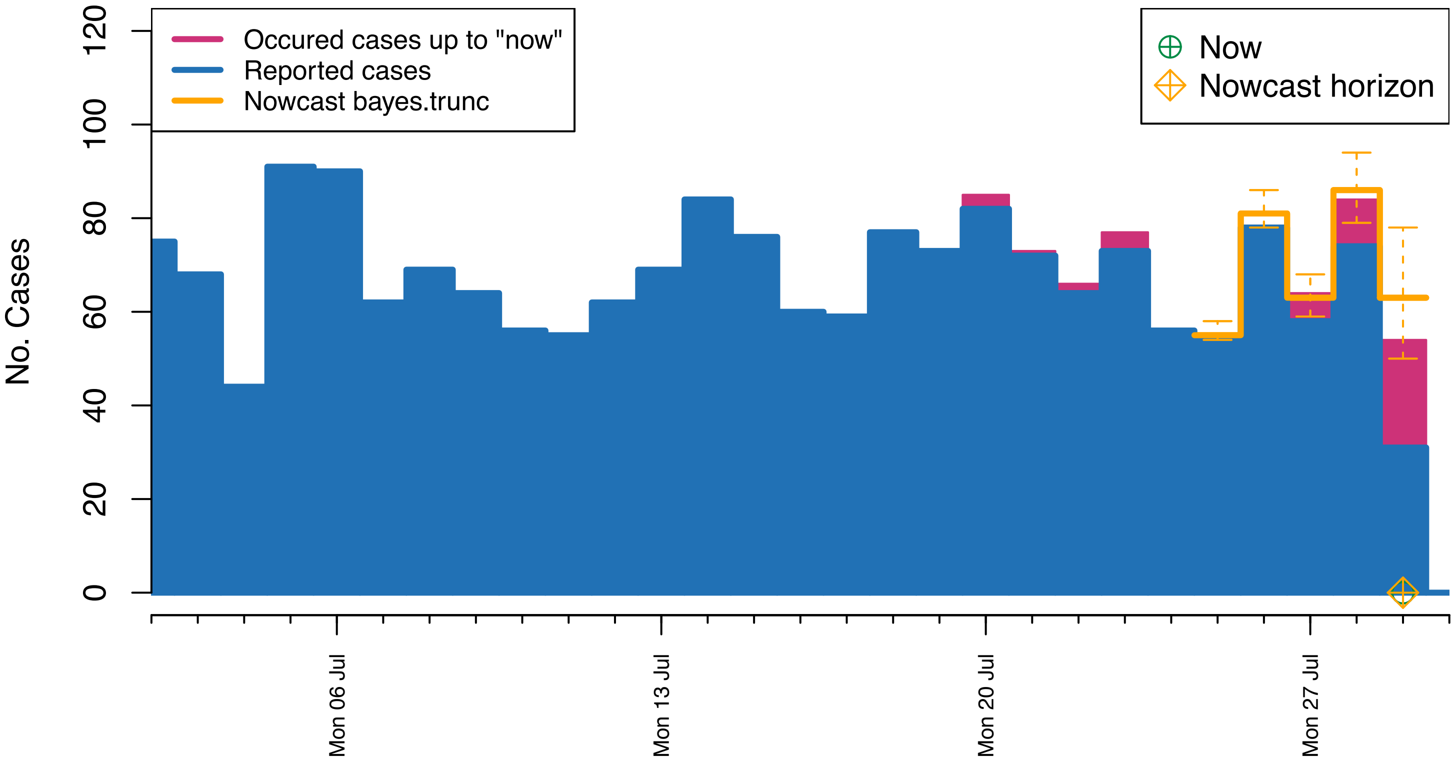

Occured cases up to "now"
Reported cases
Nowcast bayes.trunc
Now
Nowcast horizon
No. Cases
0
20
40
60
80
100
120
Mon 06 Jul
Mon 13 Jul
Mon 20 Jul
Mon 27 Jul

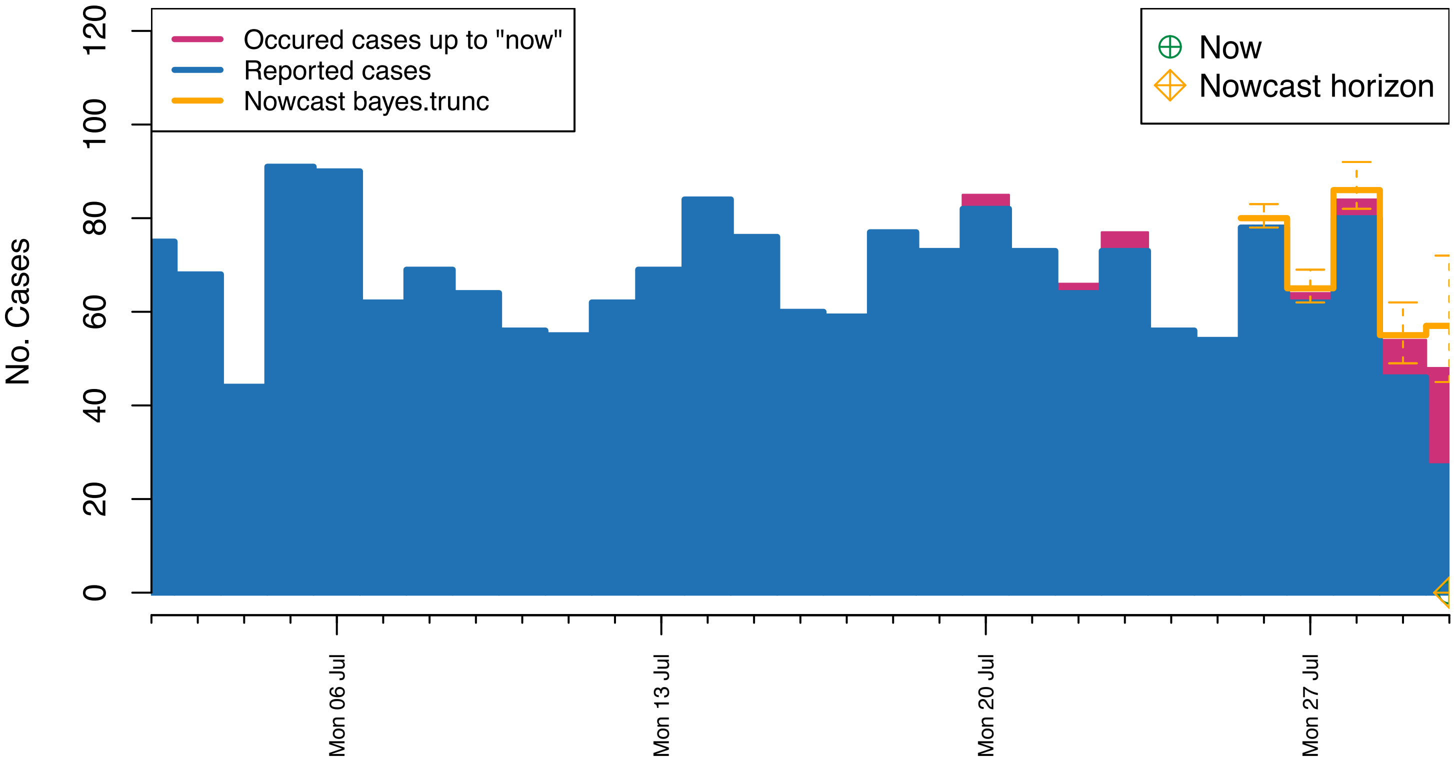

Occured cases up to "now"
Reported cases
Nowcast bayes.trunc
Now
Nowcast horizon
No. Cases
0
20
40
60
80
100
120
Mon 06 Jul
Mon 13 Jul
Mon 20 Jul
Mon 27 Jul
